# A Thickness Calibration Device Is Needed to Determine Staple Height and Avoid Leaks in Laparoscopic Sleeve Gastrectomy

**DOI:** 10.1007/s11695-015-1705-8

**Published:** 2015-05-30

**Authors:** Rose Huang, Michel Gagner

**Affiliations:** Boehringer Laboratories, LLC, Phoenixville, PA USA; Department of Surgery, Hospital Du Sacre Coeur, Montreal, QC Canada

**Keywords:** Sleeve gastrectomy, Thickness, Stapler, Reloads, Bleeding

## Abstract

**Background:**

Leaks after sleeve gastrectomy (SG) may be due to a mismatch between staple height and tissue thickness. The aim of this study was to determine the range of gastric thicknesses in three areas of stapling.

**Methods:**

SG was performed using a 40-Fr suction calibration system 4 cm from the pylorus. Measurement of combined gastric walls was accomplished with an applied pressure of 8 g/mm^2^ on the fundus, midbody, and antrum.

**Results:**

We enrolled 26 SG patients (15 women, 11 men; mean age 36.8 years). Body mass index (BMI) averaged 45.3 kg/m^2^ overall, 44.7 kg/m^2^ for males and 45.7 kg/m^2^ for females. Although male patients had a thicker stomach antrum than female patients (3.12 vs. 3.09 mm), the midbody (2.57 vs. 3.09 mm) and proximal areas (1.67 vs. 1.72 mm) were thicker in female patients. However, some maximum fundus thicknesses were up to 2.83 mm in females and 2.28 mm in males. Some antra were as thick as 4.07 mm in females and 5.39 mm in males. Also, men had a longer average staple line (22.95 vs. 19.90 cm).

**Conclusion:**

Because of the range of gastric thicknesses, a single staple height cannot be used to appose the full range of gastric wall thicknesses without potentially causing necrosis or poor apposition. To help avoid leaks, a thickness calibration device is needed to determine correct staple height.

## Introduction

The current clinical practice of selecting a staple cartridge can be categorized into two methods: singular and variable. In the singular method, the surgeon uses only one type of staple cartridge (typically black) to create the entire sleeve. However, this method risks bleeding or leaking if the thickness of the stomach is outside the indicated range of the cartridge. In the variable method, the surgeon starts with the thickest load and then chooses subsequent staple loads based on how the tissues feel. The downfall of this tactile-feedback method is its subjectivity. Without an objective measurement, an incorrect staple height may be chosen and lead to incomplete staple formation, leakage, or bleeding. Reported rates of leakage and hemorrhage following bariatric procedures employing stapling devices vary between 0 and 4.4 % and between 0.4 and 4 %, respectively [1].

A quick keyword search of *stapler*, *thickness* on the U.S. Food and Drug Administration (FDA) Manufacturer and User Facility Device Experience (MAUDE) database returned more than 200 stapler-related adverse events that questioned whether the tissue thickness exceeded the indicated range of selected staple size [2]. Each stapler manufacturer provides specific instructions regarding the appropriate tissue thickness for different anatomical locations, but because of the wide range of thicknesses in patients, there is a greater chance of error when the surgeon estimates the appropriate staple height.

The accuracy of staple reload selection has not been thoroughly studied because there is no current technology providing an objective intraoperative measurement of tissue thickness prior to cartridge selection. Given that leaks are most common near the gastroesophageal junction, where the tissue is the thinnest and where most leaks are caused by mechanical/tissue issues [3], we hypothesized that the mismatch between staple height and tissue thickness would lead to leaks after laparoscopic sleeve gastrectomy (SG). The objectives of the present study were to determine the range of gastric thicknesses in three areas of stapling and to analyze the accuracy of current tactile feedback technique in selecting the appropriate staple cartridge.

## Materials and Methods

This was a single-center, single-surgeon prospective study. Informed consent was obtained from all individual participants included in the study. Primary SG was performed with either a 40-Fr bougie or a suction calibration system (ViSiGi 3D™, Boehringer Labs, LLC, Phoenixville, PA, USA) in alternating order. The sex, age, body mass index (BMI), and comorbidity information were recorded for each patient.

**Fig. 1:**
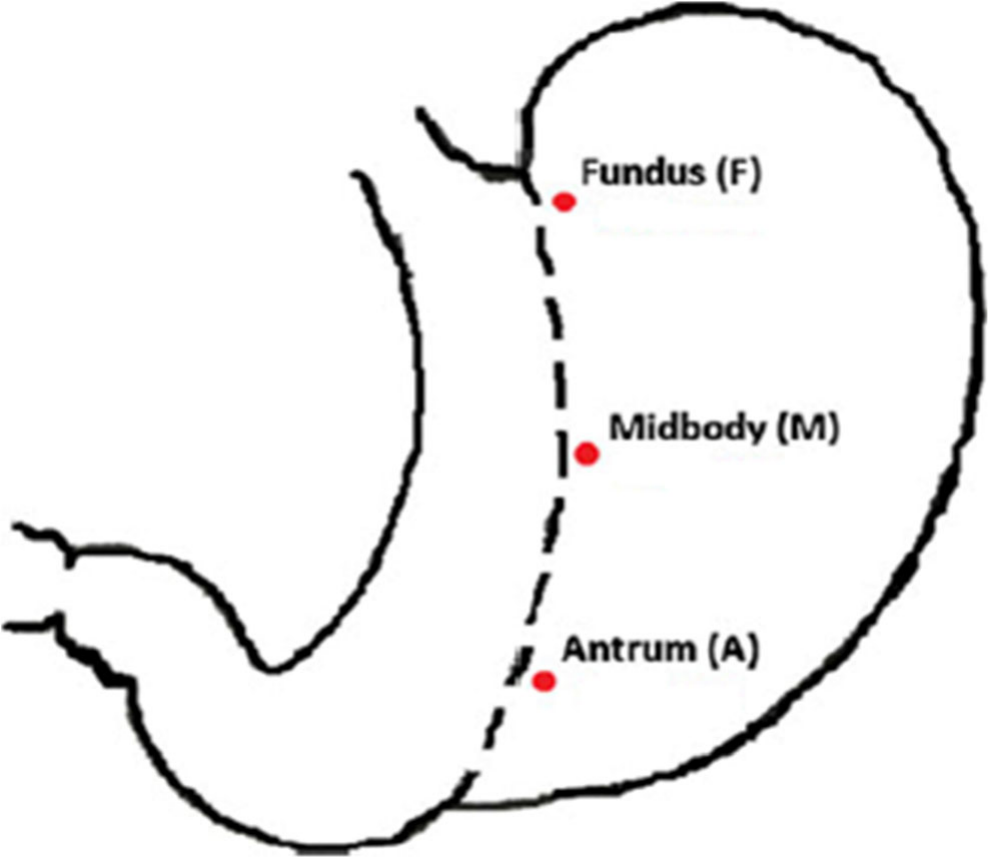
Description of the three predetermined measurement locations. Fundus, 1 cm caudal to the edge of the fundus, 0.5 cm from the staple line; midbody, halfway between the fundus and the pylorus along the staple line, 0.5 cm from the staple line; antrum, 2 cm caudal to the pylorus along the staple line, 0.5 cm from the staple line. *A* antrum, *F* fundus, *M* midbody

The method of measuring tissue thickness was modeled after the study by Elariny et al. [4]. Upon completion of laparoscopic SG, the double wall thickness of the excised gastric specimen was measured at three predetermined locations (Fig. 1).

**Fig. 2:**
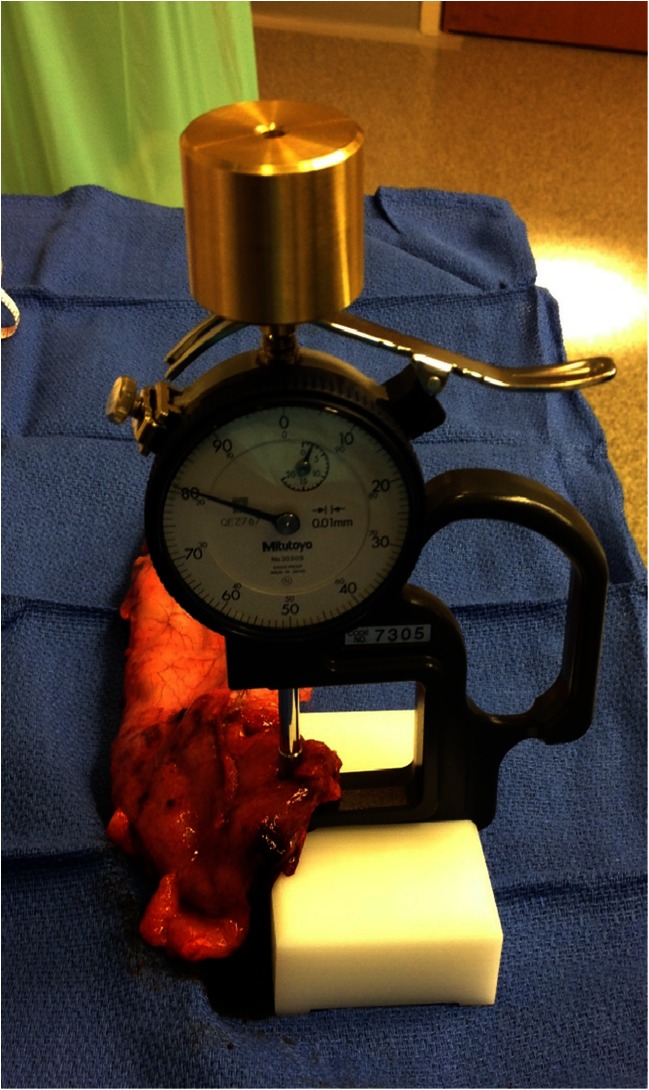
Thickness measuring device

The thickness measuring apparatus (Mitutoyo QEZ767 No. 2050S; Mitutoyo Corp., Kawasaki, Japan) (Fig. 2) was purchased online. The apparatus was modified with a weight to achieve an applied pressure of 8 g/mm^2^, which, in experiments with canine and human cadavers, was suggested by Elariny et al. to be the optimal pressure, causing negligible structural modifications and not leading to lasting complications [4]. After measuring the length of the staple line with a flexible ruler, the tissue thickness of each location was recorded after 15 s of compression [5].

### Statistical Analysis

Statistical analysis was performed with two-sample *t* test assuming unequal variances. In addition, analysis of covariance (ANCOVA) was used to determine if sex had an effect on the total length of the stomach.

To analyze the suitability of each color of staple cartridge at each predetermined anatomical location, information on closed staple height was referenced from two stapler manufacturers, Ethicon [6] and Covidien [7]. Each manufacturer publishes only one data point rather than a range of appropriate thicknesses. For this statistical analysis, each color of cartridge was assumed to be able to cover a range of thicknesses between the data points of the preceding and following cartridges (Tables 1 and 2).

**Table 1:** Published closed staple height and assumed appropriated range (Ethicon)

Color	Min closed height range (mm)	Published closed height (mm)	Max closed height range (mm)
Gray	0.5	0.75	0.875
White	0.875	1.0	1.25
Blue	1.25	1.5	1.65
Gold	1.65	1.8	1.9
Green	1.9	2.0	2.15
Black	2.15	2.3	2.5

**Table 2:** Published closed staple height and assumed appropriated range (Covidien)

Color	Min closed height range (mm)	Published closed height (mm)	Max closed height range (mm)
Gray	0.5	0.75, 0.75, 0.75	1.0
Tan	0.5	0.75, 1.0, 1.25	1.5
Purple	1.0	1.25, 1.5, 1.75	2.0
Black	1.5	1.75, 2.0, 2.25	2.5

Using the assumed range of thicknesses for each cartridge color, a normal distribution graph was generated to determine the probability of using this color at a particular anatomical location based on the measured mean thickness and standard deviation of that location. The statistical model is

$$ \left.P\left({x}_{\min }<x<{x}_{\max },\mathrm{color}\right)\right)=\frac{1}{\sqrt{2\pi \sigma {\left(\mathrm{sex},\;\mathrm{location}\right)}^2}}{e}^{-\frac{{\left(x-\mu \left(\mathrm{sex},\;\mathrm{location}\right)\right)}^2}{2\sigma {\left(\mathrm{sex},\;\mathrm{location}\right)}^2}} $$

Where *x*_min_ and *x*_max_ constitute the range of thicknesses of that color class, *σ*(sex, location) is the standard deviation of thickness of that sex population at one of the three predetermined anatomical locations, and *μ*(sex, location) is the mean thickness of that sex population at one of the three predetermined anatomical locations.

## Results

Enrollment of 26 patients (15 female, 11 male; mean age 36.8 years (range 14–74 years)) was completed in 2014. The mean BMI was 45.3 kg/m^2^ (range 35.0–61.0 kg/m^2^) overall, 44.7 kg/m^2^ (range 35.0–60.6 kg/m^2^) for males, and 45.7 kg/m^2^ (range 39.0–61.0 kg/m^2^) for females. The length of the specimen was measured along the staple line in all patients and averaged 21.2 cm (range 17.0–30.0 cm). ANCOVA demonstrated sex to be significantly correlated with the length of the stomach, which was significantly longer in males (22.9 cm (range 19.0–30.0 cm)) than in females (19.9 cm (range 17.0–26.0 cm)) (*p* < 0.001).

**Fig. 3:**
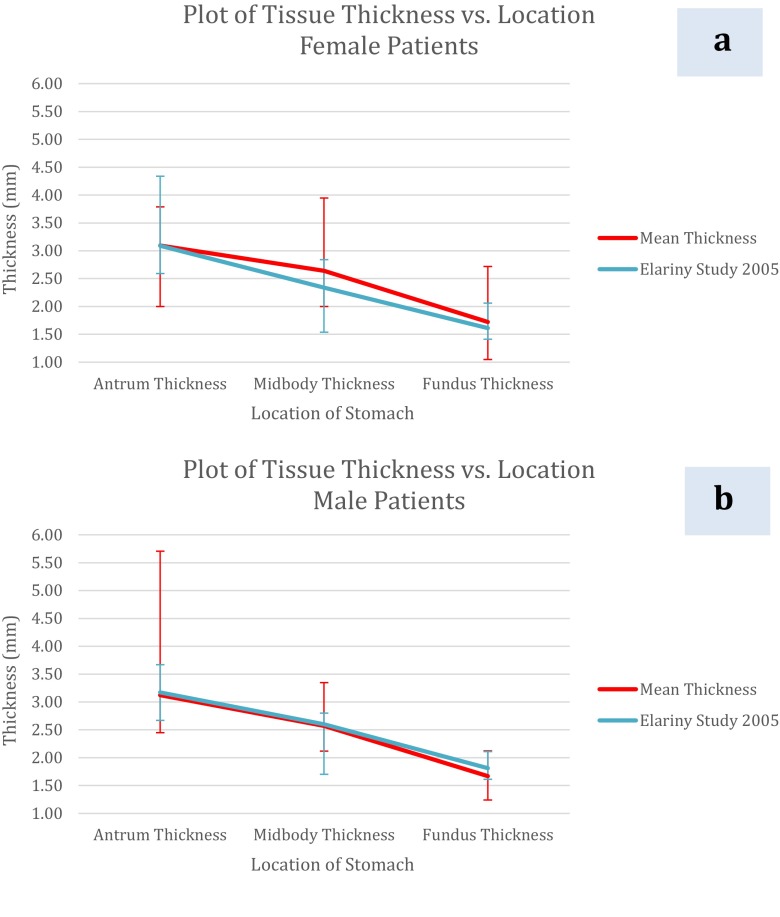
Plots of thickness vs. location for **a** female and **b** male patients in the present study and the Elariny study

Tissue thickness was measured at the three predetermined locations (antrum, midbody, and fundus) along the excised gastric specimen, as modeled after the Elariny study [4]. Figure 3 and Table 3 present descriptive data by sex and location along the gastric specimen. In both sexes, the stomach was thickest near the pyloric antrum and thinnest near the fundus, which was considered the general thickness profile.

**Table 3:** Summary statistics for tissue thickness

	Antrum thickness (mm)	Midbody thickness (mm)	Fundus thickness (mm)
Female (*N* = 15)			
Mean ± SD	3.09 ± 0.62	2.64 ± 0.60	1.72 ± 0.59
Mean ± SD (Elariny)	3.09 ± 0.553	2.34 ± 0.349	1.61 ± 0.279
Min	2.00	2.00	1.05
Max	4.07	4.00	2.83
Quartile 1–25th %	2.63	2.23	1.32
Quartile 2–50th %	3.10	2.50	1.50
Quartile 2–75th %	3.53	2.88	2.03
Male (*N* = 11)			
Mean ± SD	3.12 ± 0.81	2.57 ± 0.42	1.67 ± 0.32
Mean ± SD (Elariny)	3.17 ± 0.324	2.6 ± 0.391	1.81 ± 0.453
Min	2.45	2.12	1.24
Max	5.39	3.46	2.28
Quartile 1–25th %	2.72	2.29	1.37
Quartile 2–50th %	2.92	2.45	1.65
Quartile 2–75th %	3.21	2.82	1.85

*SD* standard deviation

However, there were five outlier cases in which the data did not follow the general thickness profile. In two patients (one male and one female), the tissue thicknesses were equal at the midbody and antrum. In two other patients (one male and one female), the tissue thickness was thicker at the midbody than at the antrum. In one female patient, the fundus was thicker than the midbody (Table 4).

**Table 4:** Summary finding for outlier cases

Sex	Cases	Fundus thickness (mm)	Midbody thickness (mm)	Antrum thickness (mm)
F	Midbody = antrum	1.52	2.00	2.00
M	Midbody = antrum	1.80	2.50	2.50
F	Midbody > antrum	2.50	2.75	2.60
M	Midbody > antrum	2.28	3.46	2.45
F	Fundus > midbody	2.80	2.60	3.60

### Analysis of Staple Cartridge Suitability with Covidien Endo GIA™ Reloads with Tri-Staple™ Technology (Covidien, Dublin, Ireland)

The suitability of a particular cartridge for a location depends on the tissue thickness at that location. In this study, the surgeon used a black cartridge at the antrum of every patient. Using the statistical model for the female patient population,

$$ \begin{array}{c}\hfill {x}_{\min}\left(\mathrm{black},\ \mathrm{Covidien}\right)=1.5\ \mathrm{mm}\hfill \\ {}\hfill {x}_{\max}\left(\mathrm{black},\ \mathrm{Convidien}\right)=2.5\ \mathrm{mm}\hfill \\ {}\hfill \mu \left(\mathrm{sex},\ \mathrm{location}\right)=\mu \left(\mathrm{Female},\ \mathrm{Antrum}\right)=3.09\ \mathrm{mm}\hfill \\ {}\hfill \sigma \left(\mathrm{sex},\ \mathrm{location}\right)=\sigma \left(\mathrm{Female},\ \mathrm{Antrum}\right)=0.62\ \mathrm{mm}\hfill \end{array} $$

**Fig. 4:**
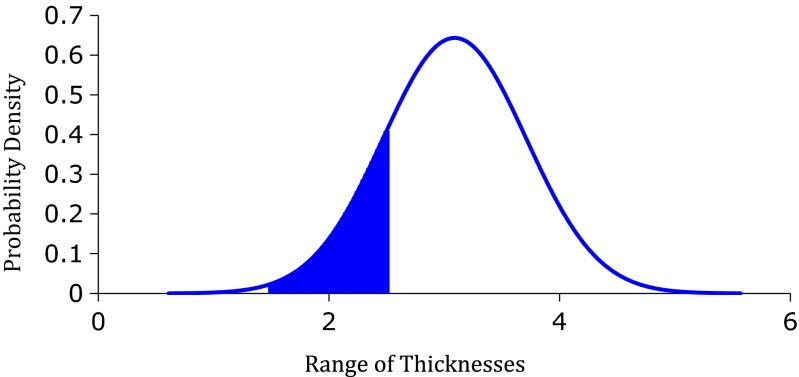
Normal distribution of the use of the Covidien black cartridge at the antra of females

The probability *P*(*x*_min_ < *x* < *x*_max_, black), i.e., the suitability of using the Covidien Tri-Staple™ black cartridge at the antrum for every female patient in this study (Fig. 4), is 16.55 %. Because the mean thickness of the antrum in females was much higher than that of the published closed staple height (3.09 vs. 2.25 mm), the use of the Covidien Tri-Staple ™ black cartridge at the antrum was appropriate in only one out of six female patients.

**Fig. 5:**
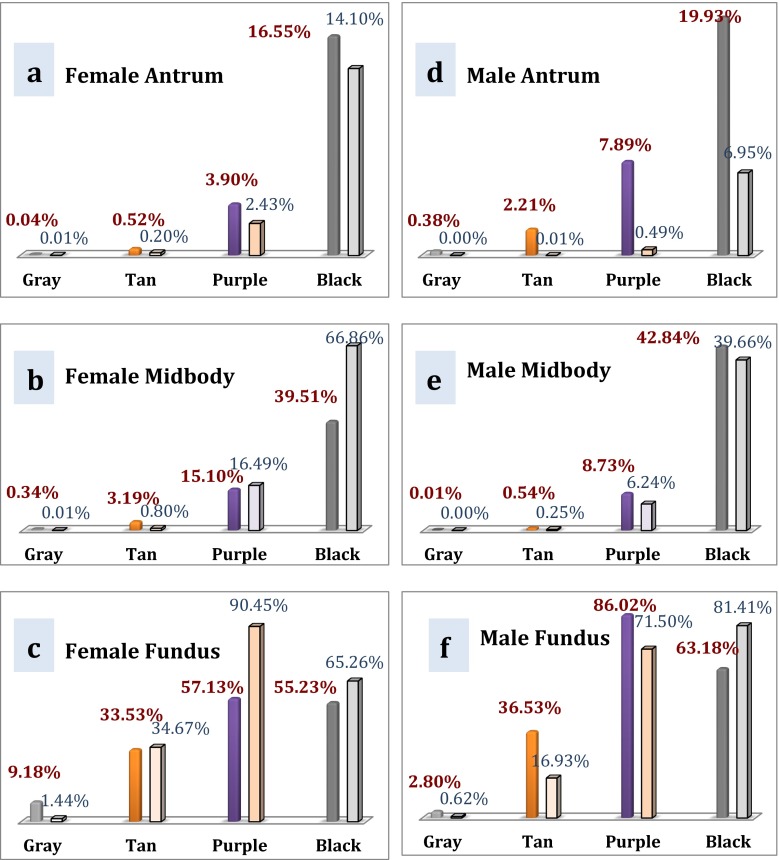
Suitability of particular color cartridges with the Covidien stapler at female **a** antrum, **b** midbody, and **c** fundus and male **d** antrum, **e** midbody, and **f** fundus

The same analysis was applied to all other Covidien Tri-Staple ™ color cartridges at the midbody and fundus (Fig. 5a–c). For example, a purple, tan, or gray cartridge at the antrum in female patients was not particularly suitable (3.90, 0.52, and 0.04 %, respectively). Using the same method, the most appropriate color cartridge at the female midbody was also black (39.51 %). Interestingly, the appropriate choice of cartridge at the fundus could be either black or purple (55.23 and 57.13 %, respectively). However, patient demographics from the Elariny study demonstrated the purple cartridge to be more suitable than the black cartridge (90.45 vs. 65.26 %) at the fundus in female patients [4]. Similarly, in the male patients in the present study, the highest probabilities for appropriate cartridge color at the antrum, midbody, and fundus were black (19.9 %), black (42.84 %), and purple (86.02 %), respectively (Fig. 5d–f), whereas for the male patients in the Elariny study, the black cartridge was more suitable than the purple (81.41 vs. 71.50 %) at the fundus. This variation in cartridge suitability at the fundus underscores the need for surgeons to be certain of the tissue thickness before choosing a cartridge.

### Analysis of Staple Cartridge Suitability with the Ethicon Echelon™ Stapler (Echelon Flex™ Endopath® Stapler; Ethicon Endo-Surgery, Inc., Cincinnati, OH, USA)

**Fig. 6:**
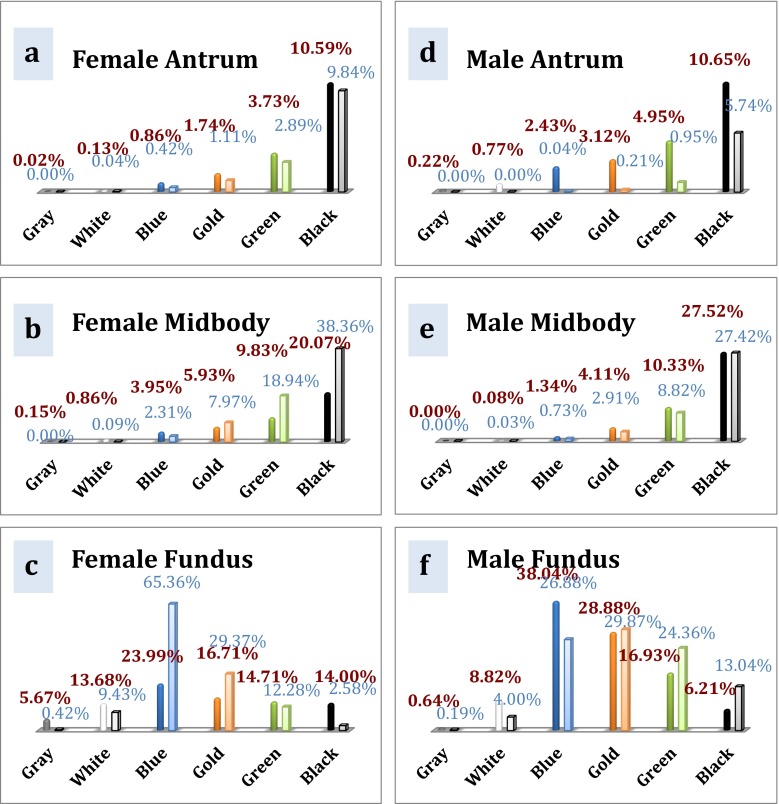
Suitability of particular color cartridges with the Ethicon stapler at female **a** antrum, **b** midbody, and **c** fundus and male **d** antrum, **e** midbody, and **f** fundus

The appropriate color cartridges at the antrum, midbody, and fundus of the female patients in the present study were black (10.59 %), black (20.07 %), and blue (23.99 %), respectively (Fig. 6a–c). Findings for the same patient population in the Elariny study were similar (black (9.84 %), black (38.36 %), and blue (65.36 %), respectively) [4].

For the male patients in our study, the appropriate color cartridges at the antrum, midbody, and fundus were black (10.65 %), black (27.52 %), and blue (38.04 %) (Fig. 6d–f); these results were inconsistent with those from the Elariny study, in which the appropriate cartridge choice at the fundus was either gold or blue (29.87 vs. 26.88 %, respectively) [4].

## Discussion

Because of a wider range of thickness coverage (Table 1), the suitability of the Covidien Tri-Staple™ initially appeared greater. However, because the three rows of the Tri-Staple™ cartridge are different heights, there is a chance that only one or two out of the three rows are appropriate for a given tissue thickness, which could compromise the integrity of the staple line. Overall, one stapler manufacturer is not superior to another. Even though the suitability might be less than 20 % at the antrum, the black cartridge is the thickest reload choice for both manufacturers on the current market. Stapler manufacturers should develop a cartridge that accommodates tissues that exceed the range of a black cartridge.

In this study and the Elariny study, the average and standard deviation for tissue thickness indicated that the black cartridge is the most suitable at the midbody. However, the location of the midbody varied with the length of the stomach. This finding warrants an objective method to measure tissue thickness intraoperatively to determine the best choice of staple reload at each staple location.

Even though each of the 26 patients provided three data points, a limitation of the present study was its small patient population. Larger, multicenter studies are needed to investigate the relationship between improper staple height and leak rate. Ideally, surgeons would measure the intact stomach tissues’ thickness prior to each staple firing. In our study, only excised stomach tissues were accessible due to the size and intended use of the tabletop thickness measuring device (Fig. 2). The effect of compression and devascularization on the thickness of the excised stomach should be taken into consideration when analyzing these data points. Transecting gastric tissue with a stapler leads to temporary inflammatory response, which caused localized edema. At the same time, the excised stomach became ischemic due to devascularization. After extracting the specimen through the 12-mm trocar site, the excised stomach was compressed again for 15 s with standardized weight on the measuring device to expel excess fluid.

There is no one-size-fits-all method for choosing the correct cartridge. When the mean and range of tissue thicknesses for our study and the Elariny study are plotted on the same graph (Fig. 3), it can be seen that the mean thickness at each location is not an overall indicator for the wide range of thicknesses we encountered. For example, gastric thickness at the fundus of females ranges from 1.05 to 2.83 mm. According to the published closed staple height information (Table 1), the last staple cartridge used could be blue, gold, green, or black for the Ethicon Echelon™ stapler or tan, purple, or black for the Covidien Endo GIA™. However, 81 % of the panel experts of the International Consensus Summit for Sleeve Gastrectomy believe that “it is not appropriate to use staples with a closed height less than that of a blue load (1.5 mm) on any part of the sleeve gastrectomy” [8]. Therefore, it is not sufficient to base recommendations for appropriate cartridges for a given tissue thickness on a single data point, and this discrepancy underscores the need for an objective means for measuring thickness.

The bariatric surgical community continues to investigate the underlying causes of leaks after SG. Current published leak rates after SG is about 2.4 % [9]. With staple mismatch being one of the causative factors, surgeons are implementing various measures such as buttressing material, fibrin sealing, oversewing, and metal clips to reduce the leak rate. These techniques, however, require additional steps as well as operating room time and cost. If buttressing material is used, its thickness should be accounted for when selecting a staple cartridge. For example, GORE® SEAMGUARD® Bioabsorbable Staple Line Reinforcement (W.L. Gore & Associates, Inc., Flagstaff, AZ, USA) measures 0.16 mm per side. When using this material, the operating surgeon would adjust the choice of cartridge by adding 0.32 mm to the perceived thickness [10].

## Conclusion

There is a need for a laparoscopic tool that accurately and quantitatively measures the thickness of stomach tissue intraoperatively. A difference of less than 1 mm in tissue thickness can be difficult to discern by touch but can make a difference in staple cartridge selection. The outlier cases in the present study demonstrate the risk of choosing an inappropriate staple cartridge solely on the basis of the general thickness profile.

A thickness measuring device would be a step toward standardizing surgical technique in laparoscopic SG. In light of stapler-related adverse events reported to Center for Devices and Radiological Health, surgeons would be able to justify the choice of cartridge by recording the measured thickness of the tissue in the patient chart, thus preventing potential lawsuits. Selecting the correct staple height does not eliminate the risk of leaks, but the operating surgeon can take on an active role in leak prevention by reducing bleeding and tissue ischemia.
